# Autologous Stem Cell Therapy: How Aging and Chronic Diseases Affect Stem and Progenitor Cells

**DOI:** 10.1089/biores.2014.0042

**Published:** 2015-01-01

**Authors:** Anastasia Yu. Efimenko, Tatiana N. Kochegura, Zhanna A. Akopyan, Yelena V. Parfyonova

**Affiliations:** ^1^ Department of Biochemistry and Molecular Medicine, Faculty of Medicine, Lomonosov Moscow State University, Moscow, Russian Federation.; ^2^ Laboratory of Angiogenesis, Russian Cardiology Research and Production Complex, Moscow, Russian Federation.

## Abstract

During recent years different types of adult stem/progenitor cells have been successfully applied for the treatment of many pathologies, including cardiovascular diseases. The regenerative potential of these cells is considered to be due to their high proliferation and differentiation capacities, paracrine activity, and immunologic privilege. However, therapeutic efficacy of the autologous stem/progenitor cells for most clinical applications remains modest, possibly because of the attenuation of their regenerative potential in aged patients with chronic diseases such as cardiovascular diseases and metabolic disorders. In this review we will discuss the risk factors affecting the therapeutic potential of adult stem/progenitor cells as well as the main approaches to mitigating them using the methods of regenerative medicine.

## Introduction

Cardiovascular diseases (CVD), particularly coronary artery disease (CAD), are the most frequent causes of mortality worldwide, and along with metabolic pathologies, especially diabetes mellitus type 2 (T2DM), they approach an epidemic status.^1,2^ An ongoing high frequency of CVD is caused both by the progressive aging of the population and an unhealthy lifestyle associated with risk factors such as obesity, hyperglycemia, hyperlipidemia, and arterial hypertension, which promote early development of atherosclerosis and progression of cardiovascular pathologies.^3^

Aging is characterized by numerous morphological and functional changes within different tissues and organs. The elasticity of blood vessels declines with age along with an increase in their stiffness, which predetermines the progression of arterial hypertension. As people age, their adipose tissue mass increases, while their muscle volume decreases, leading to the development of insulin resistance, the most important pathogenic factor of T2DM. Aging is also associated with comorbidities, the simultaneous presence of two or more different diseases, often with chronic long-lasting progression. The most frequent age-associated comorbidities confounding each other are CAD and T2DM and obesity, arterial hypertension, and T2DM.^4,5^

The target affected by the most CVD risk factors is the blood vessel wall. Endothelial dysfunction is considered to be the key pathogenic mechanism of angiopathies associated with CAD and T2DM. It should be noted that endothelial dysfunction develops as a result of the interaction of different risk factors, such as insulin resistance, hyperglycemia, and dyslipidemia. The long-term presence of these factors affects endothelial cells and promotes their apoptosis, which leads to the nitric oxide (NO) production failure. As a consequence, the vasodilatation and anti-aggregation functions of the endothelium are dysregulated along with its ability to inhibit smooth muscle cell proliferation. These factors potentiate atherosclerosis progression, forming the morphological basis of CAD.^6^

Hyperglycemia is the main pathogenic factor of T2DM, but it also underlies CVD. First, it accelerates the progression of atherosclerosis and macroangiopathies; second, it affects the microvasculature network. This effect is manifested particularly in the disturbance of neovascularization processes in different tissues. In the retina, excessive angiogenesis leads to leaky vessels and diabetic retinopathy, while in the skeletal muscles and myocardium, adaptive angiogenesis is insufficient, which promotes the development of severe ischemia, persistent trophic ulcers, and amputations.^7,8^

Despite the obvious success in treating CAD and T2DM, all available therapeutic approaches are intended to restrict disease progression. A novel promising approach for the treatment of ischemic diseases is cell-based therapy using autologous stem/progenitor cells.^9–14^ Several types of cells are involved in the neovascularization processes of ischemic tissues. Endothelial cells; their circulating progenitors (endothelial progenitor cells [EPCs]), which mobilize from the bone marrow as a response to the ischemia and vessel damage; and progenitor cells located within the vessel wall, including multipotent mesenchymal stem/stromal cells (MSCs), interact with each other and contribute to vasculature repair and regeneration.^15–17^

MSCs, derived from the bone marrow, adipose tissue (ADSCs), or other tissues, are considered a promising tool for cell-based therapy due to their high proliferative and differential potency, ability to stimulate the growth of new blood vessels and nerves, and especially their production of multiple cytokines and growth factors. These cells also secrete plasminogen activators and matrix metalloproteases (MMPs), which actively participate in the remodeling of extracellular matrix (ECM) and the proteolytic release and activation of some growth factors sequestered in the ECM. MSCs serve as a component of the vessel wall in all tissues and play an essential role in the vasculature network development and support in both normal and pathological conditions.^18^

Many types of stem/progenitor cells, including MSCs, have already been used in clinical trials of cell therapy for ischemic pathologies,^12–14,19–22^ and their safety and feasibility have been demonstrated, but the clinical effectiveness of these protocols was relatively modest and could not corroborate the promising results of preclinical studies (Table 1). One reason for the insufficient effectiveness of autologous cell therapy may be a lack of understanding about stem/progenitor cells properties in patients with CVD. Most data regarding the regenerative potential of these cells were obtained from cells derived from relatively healthy young donors. However, aging and disease itself may negatively affect stem/progenitor cells and their microenvironment, and impaired stem/progenitor cell functional properties may diminish the effectiveness of autologous cell therapy in aged patients with CAD and metabolic disorders. In this review, we analyze how aging and chronic diseases such as CAD and T2DM affect the properties of stem/progenitor cells.

**Table 1.** Meta-Analyses of Autologous Cell Therapy Clinical Trials for the Treatment of Ischemic Diseases

Disease	Reference	Design	Cell type (method of injection)	Follow-up duration	Outcome
Peripheral arterial disease	Fadini et al.^111^	37 clinical trials (6 controlled and 31 noncontrolled), *n*=701	BMSCs, PB-MNCs mobilized by G-CSF, CD34^+^ cells (IM or IA)	6 months	Improvement of ischemia surrogate indexes:
					- ABI 0.46±0.04 vs. 0.63±0.04 (*p*=0.011);
					- TcO2 22.8±2.8 vs. 35.8±2.9, (*p*=0.0002);
					- walking capacity 75.7±19.4 vs. 402.3±70.9 m, (*p*<0.0001);
					- better ulcer healing (OR 3.54, 95% CI 1.09−11.51, *p*=0.032);
					- benefit in amputation rate (OR for amputation 0.09; 95% CI 0.02–0.44, *p*=0.0005).
AMI	Clifford et al.^112^	33 RCTs, *n*=1765	BMSCs (IC)	<12 and 12–61 months	No statistically significant changes in the incidence of mortality (RR 0.70, 95% CI 0.40–1.21) or morbidity (re-infarction, hospital re-admission, restenosis, and target vessel revascularization).
					In short-term follow-up improvement: LVEF (WMD 2.87, 95% CI 2.00–3.73);
					In long-term follow-up improvement:
					- WMD 3.75, 95% CI 2.57–4.93;
					- reduce LVESV and LVEDV and infarct size;
					- positive correlation between BMSC cell dose and the effect on LVEF
Different diseases, including ischemic stroke, AMI, et al.	Lalu et al.^113^	36 clinical trials (8 RCTs), *n*=1012	MSCs (IV or IA)	0–60 months	Safety of the therapy regarding the short-term and long-term adverse effects.
					There was a significant association between MSCs injection and transient fever (OR 16.82, 95% CI 5.33–53.10).
CAD	Fisher et al.^114^	9 RCTs, *n*=659	BMSCs (mobilized CD34^+^ cells) (NOGA system)	6–12 months	Benefits:
					- reduced risk of mortality (RR 0.33; 95% CI 0.17 to 0.65; *p*=0.001);
					- improvement in angina class (MD- 0.55; 95% CI −1.00 to −0.10; *p*=0.02)
					- fewer angina episodes per week (MD −5.21; 95% CI −7.35 to −3.07; *p*<0.00001);
					- improved quality of life (*p*=0.002);
					- improved exercise/performance (*p*=0.002);
					- increase of LVEF (MD 3.47; 95% CI 1.88–5.06, *p*=0.00002).
AMI	Delewi et al.^115^	24 RCTs, *n*=1624	BMSCs (IC)	6–12 months	In 6 month:
					- improvement of LVEF 2.23% (95% CI 1.00–3.47; *p*<0.001);
					- reduction in LVESV −4.81 mL (95% CI −7.86 to −1.76; *p*<0.001);
					In 12 month:
					- improvement of LVEF (11 studies) 3.91% (95% CI 2.56–5.27; *p*<0.001);
					- reduction in LVESV −9.41 mL (95% CI −13.64 to −5.17; *p*<0.001);
					- decrease in recurrent AMI (RR 0.44, 95% CI 0.24–0.79; *p*=0.007);
					- hospital re-admission due to heart failure, unstable angina or chest pain (RR 0.59, 95% CI 0.35–0.98, *p*=0.04).
Critical limb ischemia	Benoit et al.^116^	45 clinical trials(7 RCTs), *n*=1272	BMMNCs/PBMNCs (IM)	1–48 months	Safety: low incidence of adverse events (4.2%), mortality, cancer cases are comparable with the control group.
					Benefits:
					- significantly lower amputation rate (OR 0.36, *p*=0.0004);
					- some improvement in a variety of functional and surrogate outcomes
CAD and congestive heart failure	Fisher et al.^117^	23 RCTs, *n*=1255	BMSC (IC or NOGA system)	<12 or ≥12 months	Safety: among 19 trials in which adverse events were reported, adverse events relating to BMSC treatment or procedure occurred in four individuals.
					Benefits:
					- reduced incidence of mortality (RR 0.28, 95% CI 0.14–0.53; *p*=0.0001);
					- hospital re-admission due to heart failure (RR 0.26, 95% CI 0.07–0.94; *p*=0.04);
					- reduction in LVESV (MD −14.64 mL, 95% CI −20.88 ml to −8.39 mL, *p*<0.00001);
					- improvement of LVEF (MD 2.62%, 95% CI 0.50%–4.73%, *p*=0.02);
					- reduction in NYHA functional class (MD −0.63, 95% CI −1.08 to −0.19, *p*=0.005);

RCTs, randomized controlled trials; BMSCs, bone marrow–derived stem cells; BMMNCs, bone marrow mononuclear cells; PBMNCs, peripheral blood mononuclear cells; IM, intramuscularly; IA, intra-arterial; IV, intravenously; IC, intracoronary; ABI, ankle brachial index; TCO2, transcutaneous oxygen tension; G-CSF granulocyte colony-stimulating factor; AMI, acute myocardial infarction; CAD, coronary artery disease; NYHA, New York Heart Association class; LVEF, left ventricular ejection fraction; LVESV, left ventricular end-systolic volumes; LVEDV, left ventricular end-diastolic volume; CI, confidence interval; OR, ratio of the odds; RR, relative risk; WMD, weighted mean difference; MD, mean difference.

## Influence of Aging on Stem/Progenitor Cells

### Notion of aging in stem/progenitor cell compartment

Stem/progenitor cells mediate lifelong physiological renewal and regeneration of tissues. Attenuated regeneration potential of aged organisms might be caused by age-associated changes of stem/progenitor cells activity. Both intrinsic and extrinsic mechanisms as well as cell response to systemic signals are involved in the normal and pathological aging of stem/progenitor cells, including MSCs.^23,24^ Inhibition of stem/progenitor cell functional activity might be mediated by telomere shortening and decreased telomerase activity,^25^ decline of the proliferation potency, weakening of the antioxidant protection system and/or presence of oxidative stress,^26^ irreversible protein modification, and accumulated damage to the DNA repair system and methylation pattern.^27^

It should be emphasized that substantial differences exist between cellular senescence and organismal aging. Aging can be defined as “the sum of primary restrictions in regenerative mechanisms of multicellular organisms.”^28^ This definition highlights the involvement of stem/progenitor cells in cell replenishment and thus in influencing lifespan.^28^ A wide spectrum of age-associated pathologies exist, including atherosclerosis, CAD, stroke, oncological diseases, psychiatry disorders, and so forth.

Cellular senescence is the equivalent of replicative senescence, and it can be defined as “an essentially irreversible arrest of cell division,” which underscores the changes in both function and replicative capacity of senescent cells. The senescent cell becomes a major actor of the aging process, among others, by acquiring a senescence-associated secretory phenotype.^29^

### Aging markers in stem/progenitor cells

Influence of aging on stem/progenitor cells is well studied for MSCs. Bone marrow-derived MSCs from aged donors were shown to have worse proliferation and differentiation capacity^28,30–33^ and were less effective for tissue repair after ischemic injury; for example, in an animal model of myocardial infarction.^33^ In contrast to bone marrow-derived MSCs, the number of ADSCs in fat tissue, as evaluated by flow cytometry, does not decrease with age.^34,35^ However, their clonogenic and proliferation capacity declines^36–40^ as does the differentiation potential^34,36,41^ and the production of vascular endothelial growth factor (VEGF).^42^

Using the appropriate age markers is important for the evaluation of aging impact to the properties of stem/progenitor cells. One marker is the relative telomere length, which indicates the number of cell divisions. Telomere shortening is considered to be the main causal mechanism for replicative cell senescence and age-associated telomere damage, and the diminution of the telomere “capping” function and associated p53 activation have emerged as prime instigators of tissue stem/progenitor cells functional decline.^25,43^ In poorly differentiated cells, telomere length is maintained due to the high activity of telomerase. Telomerase also has some telomere-independent functions. Telomerase activity was shown to be the highest in stem and tumor cells and was detected to a certain degree in many kinds of progenitor cells.^37,44^ Its activity is repressed as stem cells start to differentiate.^25,37^ Age-associated telomere shortening in MSCs has been shown.^28,30^ According to our data, relative telomere length decreases with age in both murine and human ADSCs.^45,46^ Interestingly, telomeres in “aged” stem/progenitor cells are still longer than telomeres in somatic cells from the same tissues,^25,27^ which could be explained by the lower proliferation activity of stem/progenitor cells or by special mechanisms of telomere-shortening prevention.

Age-dependent decrease of stem/progenitor cell proliferation activity is considered to be related to higher expression of cell cycle inhibitors like p16INK4a, p21, p53, and p19ARF or inhibition of their degradation.^28,47^ In accord with telomere shortening in ADSCs from aged patients and old mice, we observed a decrease in proliferation activity of ADSCs with age as well as fewer actively proliferating cells.^45,46^

Because stem/progenitor cells persist in tissues throughout life, albeit mostly in a quiescent state, they experience age-related long-term exposure to genotoxic insults from both endogenous and exogenous sources. Accordingly, accumulation of DNA damage in aged stem/progenitor cells has been noted in several studies. The DNA damage response pathways necessary for stabilizing the genomic integrity may have reduced activity in stem cells with age. Accumulation of DNA damage in aged stem cells could cause stem cell senescence or apoptosis and alterations in stem cell self-renewal and differentiation.^24^

Oxidative stress and its regulators play an important role in aging of stem/progenitor cells. Increasing production of reactive oxygen species (ROS) and weakening of the antioxidant protective system in cells promote oxidative damage and lead to the loss of redox control, which affects ROS-regulated biological processes such as growth, proliferation, migration, apoptosis, differentiation, and so forth.^28^ Different factors like hypoxia inducible factor-1 alpha (HIF-1α), ataxia telangiectasia mutated (ATM) protein, Bmi-1, and FoxO family factors may change ROS level in the cells.^27^ In bone marrow–derived MSCs obtained from aged patients, the activity of superoxide scavenger (superoxide dismutase) is decreased and the levels of ROS, NO, and oxidized and glycosylated proteins are increased.^30^ Supporting the hypothesis that ROS generation may promote stem cell aging, studies of aged human stem/progenitor cells, including MSCs, neural stem cells, and others, have found that excessive cellular ROS concentrations lead to abnormal proliferation, malignancy, and compromised stem cell self-renewal and differentiation capacity.^24^ The role of ROS in aging-related changes of ADSC properties was also demonstrated.^48^

A direct relationship between mitochondrial dysfunction and aging has been suggested by many studies,^49^ and an age-related deficit of mitochondrial function leading to respiratory chain dysfunction in stem/progenitor cells is actively being investigated. These effects are considered to result largely from an accumulation of mutations in mitochondrial DNA damaged by elevated ROS or other mechanisms.^24,49^ Aside from primary mitochondrial lesions (mitochondrial DNA mutations), secondary alterations in mitochondrial function driven by age-related cellular and metabolic changes may also contribute to the aging process.^49^

### Age-related changes of stem/progenitor cells paracrine functions

Paracrine activity of stem/progenitor cells, including MSCs, changes with age. Interleukin (IL)-6 secretion by bone marrow–derived MSCs co-cultured with T cells decreased in aged patients.^32^ According to our data, the angiogenic potential of ADSCs is significantly impaired during aging. We have shown that ADSCs isolated from old mice (18 months), along with expressing age markers (shorter telomeres, higher rate of apoptotic cells, less proliferative capacity, enhanced oxidative damage), have an impaired ability to stimulate blood vessel growth on *in vitro* and *in vivo* models of angiogenesis compared to ADSCs from young animals (1–2 months).^45^ Similar results were obtained with human ADSCs, including in patients with cardiovascular pathologies.^46^ We demonstrated that the mechanisms of age-associated decline of ADSC angiogenic activity include decreased production of key pro-angiogenic factors such as VEGF, placental growth factor (PlGF), hepatocyte growth factor (HGF), angiopoetin-1, and angiogenin.^46^ It should be noted that age-associated differences in the expression of pro-angiogenic factor genes were not found,^46^ indicating that posttranscriptional mechanisms, such as regulation by microRNA,^38,50^ age-associated protein misfolding, and so forth, could underlie the decreased secretion of angiogenic factors by ADSCs from aged patients.

Apart from the angiogenic growth factor effects, ECM remodeling is crucial for successful angiogenesis. ECM remodeling and direction of cell migration for vessel wall formation are regulated by multiple factors such as urokinase (uPA) and its receptor (uPAR), plasminogen activator inhibitor-1 (PAI-1), MMPs, and so forth. Analyzing the expression of factors involved in ECM remodeling and vascular cell migration and invasion, we found that mRNA levels of uPA, uPAR, and PAI-1 as well as uPAR surface expression along with the activation of MMP-2 and MMP-9 were higher in ADSCs from aged patients with CAD.^46,51^ These results are consistent with those we previously obtained from ADSCs from young and old mice.^45^ The findings could reflect the adaptive reaction of stromal cells to the age-associated ECM changes within the blood vessel wall^52^ and increasing levels of pro-inflammatory factors and ROS. Also considering the regulatory role of the uPA system in growth factor–induced endothelial cell migration and invasion and stimulation of angiogenesis in ischemic tissues,^53^ we can speculate that its activation in ADSCs from aged patients might be a compensatory response to the reduction of pro-angiogenic factor secretion.

### Aging as a risk factor for autologous cell therapy

Given the total evidence, aging could essentially affect the properties of stem/progenitors cells, thereby diminishing the effectiveness of autologous cell therapy. Testing of cell material before use may be required along with developing effective approaches for pretreatment or modification of stem/progenitor cells from aged patients to enhance their therapeutic potential. Some of these approaches will be discussed below.

Since MSC are considered to be the components of vessel wall and take part in its repair after injury, cellular modifications due to aging can be an important pathogenic factor of age-related diseases such as atherosclerosis, diabetes, and arterial hypertension.^11^ Aging is also associated with EPC dysfunction that further negatively affects the neovascularization and angiogenesis in tissues.^54^

We can conclude that organismal aging is a complex process in which resident stem/progenitor cells are involved both as a cell reservoir for the repair and regeneration of tissues and as targets exposed to the multiple local and systemic stimuli of the aged organism. These changes should be considered during the development of cell-based therapy using different types of autologous stem/progenitor cells.

## Influence of Chronic Diseases on Stem/Progenitor Cells

### Stem/progenitor cell quantity and characteristics in the presence of chronic diseases

During recent years a substantial amount of data have shown that chronic pathologies, including CAD and T2DM, affect the properties of stem/progenitor cells. It should be noted that widespread risk factors associated with CAD (age, dyslipidemia, obesity, smoking, arterial hypertension, glucose intolerance) may have an impact on the decrease in number and/or functional activity of stem and progenitor cells.^16,55–57^ Among the pathologies affecting the functionality of stem and progenitor cells, autoimmune diseases, such as systemic sclerosis and systemic lupus, may be included.^58^

Numerous studies showed that the number, proliferation activity, ability for adhesion, migration, and angiogenic properties of EPCs are significantly impaired in patients with CAD^55,59–61^ and metabolic disorders including obesity and T2DM.^62–65^ Van Ark et al.^66^ demonstrated that in patients with T2DM both EPCs and circulating angiogenic cells levels were reduced and the ratio between EPCs and smooth muscle progenitor cells was disturbed, which may translate into reduced vascular repair capacity, thereby promoting macrovascular disease in T2DM.

According to the results of several studies the number of MSCs is unlikely to be decreased in patients with cardiovascular diseases, but their regenerative potential may be attenuated. Thus, Harris et al.^35^ showed that the number of ADSCs obtained from 50 patients with different vascular pathologies was relatively consistent independent of age and comorbidities like obesity, T2DM, and so forth. Similar results were obtained by Madonna et al.^57^ in 42 patients with different grades of cardiovascular risk. However, ADSCs isolated from adipose tissue of patients with T2DM were shown to have lower proliferation activity and were less responsive to pro-angiogenic stimuli such as hypoxia.^67^ In patients with obesity, impaired differentiation potential of ADSCs and a decline in their ability to stimulate blood vessel growth were observed.^68^ In a study performed by Vecellio et al.,^69^ MSCs obtained from the heart tissue of patients with T2DM were characterized by a reduced proliferation rate, diminished phosphorylation at histone H3 serine 10 (H3S10P), decreased differentiation potential, and premature cellular senescence compared to the control group.

### Changes of stem/progenitor cell properties in patients with CAD and T2DM: evidence from ADSCs

We analyzed how ADSC properties are changed in patients with CAD and T2DM.^70^ We showed that ADSCs from the patients with CAD (*n*=32) and CAD+T2DM (*n*=28) had similar morphology and immunophenotype, preserved adipogenic and osteogenic differentiation potency, and higher proliferation activity, but shorter telomeres compared to ADSCs from control patients without established chronic pathologies (*n*=19). These findings might reflect the depletion of progenitor cell compartment in the presence of chronic pathologies such as CAD and T2DM.

Analyzing ADSCs as a tool for therapeutic angiogenesis we found that the angiogenic potential of ADSCs obtained from patients with CAD and with CAD+T2DM was decreased compared to patients without established cardiovascular pathologies independent of such factors as age and sex. We also did not see any statistically significant evidence that T2DM had an additional impact on the decline of ADSC angiogenic activity when combined with CAD. Since a paracrine mechanism is considered to be the main regulator of the beneficial effects of ADSCs, we examined the ability of ADSC to secrete some key angiogenic and anti-apoptotic growth factors. Interestingly, we revealed significantly higher production of some pro-angiogenic factors by ADSCs: VEGF and HGF for patients with CAD and HGF and PlGF for patients with CAD+T2DM, whereas the angiogenic activity of all products secreted by ADSCs from patients with both CAD and CAD+T2DM was significantly decreased compared to the control group. Absence of an elevation in the VEGF level in ADSC-conditioned medium from patients with CAD+T2DM may be associated with hyperglycemia. A high glucose level reduces endothelial nitric oxide synthase (eNOS) and stimulates inducible NOS (iNOS) expression that can inhibit *HIF-1a* gene expression. The activity of *HIF-1a* gene expression in its turn directly mediates *VEGF* gene expression. Moreover, by reducing eNOS the high glucose level inhibits the production of NO in endothelial and vascular smooth muscle cells, which is known as a downstream mediator of several angiogenic factors including angiopoietin-1.^71,72^

The process of angiogenesis depends on the intricate balance between angiogenic and angiostatic factors. We speculated that the main cause of impaired angiogenic potential of ADSCs obtained from patients with CAD and CAD+T2DM was the increased level of the angiogenesis inhibitors. Among others we analyzed production of endostatin and thrombospondin-1 (THBS1) by ADSCs and showed that *THBS-1* gene expression is significantly increased in ADSCs from patients with CAD and CAD+T2DM, but we could not confirm this finding on the protein level. However, statistical analysis revealed negative correlation between mRNA THBS-1 expression and angiogenic activity of summary products secreted by ADSCs from patients with CAD and CAD+T2DM. It allows us to assume that some indirect mechanisms of THSP-1 participation in the angiogenic effects of ADSCs could be discussed.

As for the factors involved in ECM remodeling, we found that the mRNA level of PAI-1 as well as its secretion by ADSCs was significantly increased in groups of patients with CAD and CAD+T2DM. PAI-1 is one of the primary regulators of the fibrinolytic system, and it has a crucial effect on cell migration and adhesion. A high level of PAI-1 was linked with a high risk of CAD, diabetes, and obesity. PAI-1 can both promote and inhibit vascular remodeling, but its role in angiogenesis and tissue regeneration is still controversial. The balance between these two mechanisms may depend on a disease state. Plasma PAI-1 is closely correlated with such factors as hypoxia, glucose-related signaling molecules, inflammatory cytokines, triacylglycerol, and insulin.^73–75^ Acosta et al.^76^ showed that ADSCs obtained from patients with T2DM have less fibrinolytic ability because they secrete more PAI-1 and less tissue activator of plasminogen and D-dimer. This situation led to the development of microthrombotic complications when these cells were used to treat critical low limb ischemia. PAI-1 actively interacted with uPA, an important factor of extracellular proteolysis that not only specifically cleaves plasminogen and converts it into plasmin, causing activation of different MMPs, but also initiates intracellular signaling upon binding to its receptor on the cell surface. It therefore plays multiple roles in vascular remodeling and angiogenesis. We propose that PAI-1 produced by ADSCs exerts anti-angiogenic effects also through the inhibition of uPA.

Importantly, we demonstrated that by neutralizing only one factor in ADSC-conditioned medium—PAI-1—we could partially restore the angiogenic activity of ADSCs obtained from patients with chronic diseases. Our data are corroborated by the results obtained by Tashiro et al.^77^ who have shown that PAI-1 inhibition *in vivo* under ischemic conditions increases the activity of pro-angiogenic factors such as VEGF-A and fibroblast growth factor (FGF)-2 and leads to the stimulation of angiogenesis and improved restoration of tissue perfusion. However, MMP-9 deficiency and VEGF-A blockade reversed the PAI-1 inhibitor-mediated neovascularization within the ischemic niche. Some experts have suggested that a PAI-1 blockade and an increase in *MMP-9* gene expression under ischemic conditions could be promising molecular approach in the therapeutic angiogenesis.^77,78^ It should be noted that we did not find significant increase in the MMP-9 mRNA level in ADSCs from patients with cardiovascular pathologies in our study. Therefore, we assume that high expression of PAI-1 and insufficient activity of MMP-9 and, for diabetic patients, VEGF may be one of the mechanisms of impaired angiogenic potential of ADSCs from patients with CAD and CAD+T2DM.

### Hyperglycemia affects both specialized and stem/progenitor cells

One of the main adverse factors for cells from patients with T2DM is hyperglycemia. Several studies have shown that long-term exposure of vascular cells to a high level of glucose causes dysfunction and promotes apoptosis.^79^ We have previously found that endothelial cells cultured in hyperglycemia modeling conditions (25 mM of glucose) have an impaired ability for VEGF- and serum-induced migration as well as forming capillary-like structures on Matrigel *in vitro*. To reveal the possible mechanisms of these effects we analyzed the expression of VEGF receptors (VEGFR) on endothelial cells because this growth factor specifically induced adaptive angiogenesis in ischemic tissues. We observed fivefold decrease in VEGFR1 expression and more than twofold decrease in VEGFR2 expression in the endothelial cells cultured in hyperglycemic conditions compared to the standard conditions.^80^

We also analyzed the influence of hyperglycemia on progenitor cells localized in the perivascular niche, such as MSCs. Culturing of ADSCs in high glucose (25 mM) medium didn't affect their proliferation activity, viability, or migratory properties, but the ability of the total secreted products of these cells to stimulate capillary-like tube formation *in vitro* was significantly decreased. We evaluated the transcriptome of ADSCs cultured in the standard conditions or in the presence of high glucose (25 mM) and found the most significant changes in gene expression of ephrin receptors, vitronectin, and plexin domain 1 contained protein (decreased in hyperglycemic conditions) and leptin, tumor necrosis factor α (TNFα), plasminogen, and angiopoetin-like factor 3 (increased in hyperglycemic conditions). However, we failed to find any significant changes in gene expression of key angiogenesis-related factors and their receptors associated with high-glucose culturing. To explain the decreased angiogenic activity of ADSCs cultured in hyperglycemic conditions, we focused on the elevated level of plasminogen in these cells. It is known that high expression of plasminogen in tumors is associated with activation of MMPs that perform cleave plasminogen to generate angiostatin, an angiogenesis inhibitor. This mechanism might also be realized in ADSCs, impairing their angiogenic potential. A combination of increased expression of TNFα and angiopoetin-like factor 3 also could affect ADSC angiogenic activity because these factors stimulate apoptosis of endothelial cells. Taken together, long-term incubation of vascular cells in hyperglycemic conditions affect both endothelial cells and ADSC functional properties, which can be one of the reasons for insufficient adaptive angiogenesis in patients with diabetes.^81^

It should be emphasized that EPC number and functional properties are also significantly changed in patients with metabolic disorders.^82,83^ EPCs play a very important role in the repair of damaged endothelium and the revascularization of ischemic tissues. T2DM has been shown to affect EPC mobilization from the bone marrow, resulting in a lower amount of EPCs in circulation and a distortion of angiogenesis and normal repair processes in injured tissues.^84^ This promotes the progression of ischemic diseases and adversely affects the prognosis of patients with T2DM.

No consensus exists about the EPC phenotype for the identification of these cells. In clinical practice, the most frequently used markers are CD133^+^/CD34^+^, CD133^+^/CD34^+^/VEGF-R2^+^, CD133^+^/VEGF-R2^+^, CD34^+^/VEGF-R2^+^.^85–87^ However, some investigators have demonstrated that the subpopulation identified as CD45^−^VEGFR2^+^(KDR)CD34^+^ cells consists of mostly leukocyte progenitors, particularly monocytes.^88,89^ Contradictory data about the immunophenotype of EPCs is explained by the substantial overlap of surface markers between the endothelial and hematopoetic progenitors and differences in the protocols and flow cytometry analysis.

Given the association of both CAD and T2DM with endothelial dysfunction and tissue ischemia, EPCs might serve as biomarkers of severity and prognosis in patients with such pathologies. We analyzed total populations of CD34^+^ cells, including hematopoetic stem/progenitor cells as well as EPCs in the peripheral blood of patients with CAD and with CAD+T2DM. We found that EPC numbers were significantly higher in the patients with CAD only compared to the healthy control age- and sex-matched subjects. This increase could be caused by the stimulated mobilization of these cells from bone marrow in response to tissue ischemia. But in patients with CAD+T2DM, such an elevation was not observed, suggesting the possible distortion of EPC mobilization in T2DM.

Analyzing EPC numbers based on the severity of T2DM, we found that in patients with compensated and subcompensated diabetes, the level of EPCs was similar to the normal level, but patients with decompensated diabetes and comparable severity of CAD had significantly lower numbers of these cells, which could indicate impaired mobilization of EPCs from bone marrow. We showed that EPC numbers were negatively correlated with glucose levels.^58^ Similar data were obtained by Churdchomjan et al.^84^

Altogether ample evidence exists that chronic diseases such as CAD and T2DM severely affect not only the fully terminated endothelial cells, but also stem/progenitor cells involved in vascular remodeling in normal and pathological conditions. Further investigation of the influence of the mechanisms of chronic diseases on the quantitative and functional characteristics of stem/progenitor cells is important for the development of effective methods for diagnosis and correction of micro- and macroangiopathies.

## Perspective of Personalized Approaches to Autologous Cell Therapy

Taking into account the results of numerous studies discussed, it is important to know the changes of patient's stem/progenitor cells to develop the methods of individual pretreatment of autologous cell material to improve its therapeutic potential.^90,91^

Hypoxic preconditioning is one of the most accessible and widespread approaches to treat stem/progenitor cells before transplantation to enhance their resistance to the ischemic stimuli and stimulate production of angiogenic factors in these cells. Many studies have shown that hypoxia stimulates angiogenic properties of bone marrow–derived MSCs and ADSC.^45,92–97^ Thus, culture of bone marrow–derived MSC in hypoxic conditions before transplantation improved the survival of these cells in damaged tissues as well as their ability to stimulate blood vessel growth.^98^

In several studies, including our work, even short-term (2–3 days) exposure of ADSCs to low oxygen levels (1%–5%) stimulated their proliferation and angiogenic activity.^93–97,99^ Moreover, we found that after hypoxic preconditioning the balance between pro- and anti-angiogenic factors was shifted to the more pro-angiogenic profile in ADSCs obtained from both young and aged donors. The preconditioning enhances the ability of ADSCs to stimulate angiogenesis and stabilize the newly forming vessels.^45,94^ Despite the many advantages of using hypoxic preconditioning as a pretreatment procedure for autologous stem/progenitor cells, length of hypoxia, oxygen level, and number of hypoxia/reperfusion cycles vary from study to study, so the optimal pattern of hypoxic preconditioning remains open for discussion.

For some purposes stem/progenitor cells could be initially differentiated *ex vivo* using the specific induction medium^100^ or sorted by the specific markers like CD34.^101^ Several growth factors and chemokines could be added to the culture medium of stem/progenitor cells, such as basic FGF (bFGF), epidermal growth factor, TNFα, insulin-growth factor-1, bone morphogenetic protein-2, and so forth, to enhance the viability, proliferation, migratory properties, and therapeutic potential of these cells.^101,102^

Another approach includes modulation of intracellular signal cascades, for example, by pretreating stem/progenitor cells with statins that activate the Akt/eNOS pathway within the cells^103^ or with protein kinase p38 inhibitor,^104^ or by culturing the cells in the presence of melatonin to increase their viability and proliferation activity and stimulate angiogenic factor production and angiogenic potential after transplantation to the ischemic kidney parenchyma.^105^ Lutolf et al.^106^ described a novel approach to restore the regenerative potential of aged stem/progenitor muscle cells: p38 inhibition combined with bioengineered modeling of the cell microenvironment.^106^

Sun et al.^31^ have demonstrated that MSCs from old animals could have their capability for renewal and osteogenic potential restored by being cultured on ECM produced by the cells of young donors. Similarly, EPCs isolated from old rat peripheral blood recover their functions *in vitro* and *in vivo* after being cultured in the presence of young animal's serum.^36^

One of the most promising tools for stem/progenitor cell pretreatment is genetic modification of these cells. Through the use of different vectors, various genetic constructions could be efficiently inserted to ADSCs. ADSCs modified by VEGF and HGF produced significantly higher amount of these factors and had enhanced angiogenic activity.^36,107,108^ Modification of stem/progenitor cells with other factors improved their ability for homing (stromal derived factor-1, CXCR4), increased viability (Akt, Bcl-2, heat shock protein-20, hemoxygenase-1, bFGF), and stimulated paracrine function (angiogenin, angiopoetin-1, IL-18-binding protein, TNFα receptors 1 and 2).^57,91^ Several factors could be combined for modification; for example, using MMP-3 tissue inhibitor^109^ or telomerase^110^ with VEGF to modify MSCs obtained from aged donors allowed significant improvement of cell regenerative potential.

## Conclusions

**FIG. 1.**
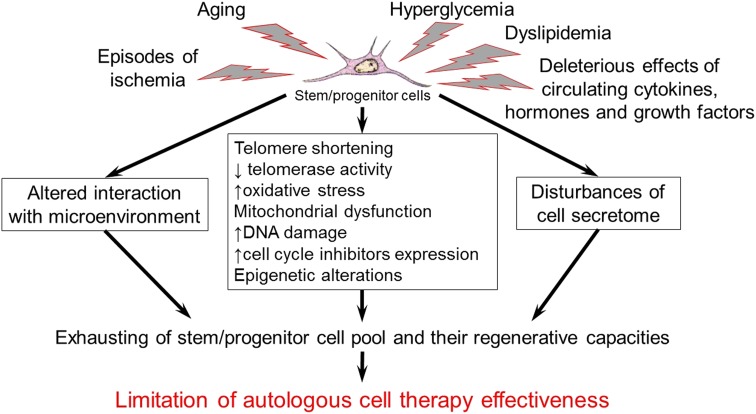
Aging and chronic diseases affect adult stem/progenitor properties and may cause low effectiveness of autologous cell therapy.

**FIG. 2.**
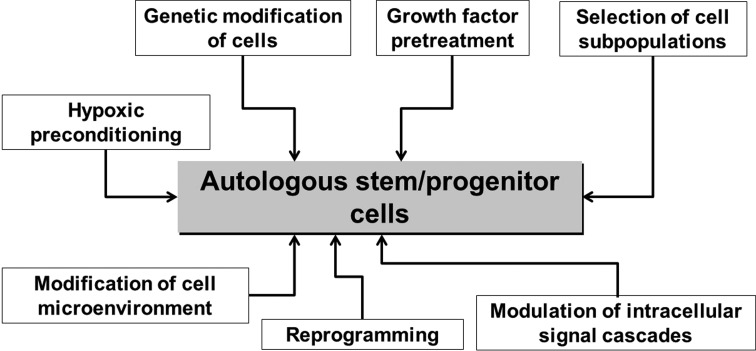
Variety of approaches for pretreatment or modification to enhance the therapeutic potential of stem/progenitor cells from aged patients with multiple comorbidities.

Aging and chronic diseases including CVD and diabetes substantially affect stem/progenitor cells of adult organism (Fig. 1). Such conditions could restrict the effectiveness of autologous cell therapy in aged patients with CAD, lower limb ischemia, T2DM and other chronic pathologies, although these patients are some of the most obvious candidates for cell therapy. These findings also indicate the necessity of careful testing of autologous cell material before use as well as developing effective approaches for pretreatment or modification of stem/progenitor cells from aged patients with multiple comorbidities (Fig. 2) to enhance therapeutic potential and stimulate endogenous regenerative processes.

## Abbreviations Used

ADSC: adipose-derived stem cell
bFGF: basic fibroblast growth factor
CAD: coronary artery disease
CVD: cardiovascular disease
ECM: extracellular matrix
eNOS: endothelial nitric oxide synthase
EPC: endothelial progenitor cell
FGF: fibroblast growth factor
HGF: hepatocyte growth factor
IL: interleukin
iNOS: inducible nitric oxide synthase
MMP: matrix metalloprotease
MSC: mesenchymal stem/stromal cell
NO: nitric oxide
PAI: plasminogen activator inhibitor
PlGF: placental growth factor
ROS: reactive oxygen species
T2DM: type 2 diabetes mellitus
TNFα: tumor necrosis factor
uPA: urokinase
uPAR: urokinase receptor
VEGF: vascular endothelial growth factor
VEGFR: vascular endothelial growth factor receptor
